# The Effect of Chromosome Arm 1BS on the Fertility of Alloplasmic Recombinant Lines in Bread Wheat with the *Hordeum vulgare* Cytoplasm

**DOI:** 10.3390/plants10061120

**Published:** 2021-05-31

**Authors:** Nataliya V. Trubacheeva, Mikhail G. Divashuk, Anastasiya G. Chernook, Igor A. Belan, Ludmila P. Rosseeva, Lidiya A. Pershina

**Affiliations:** 1 Institute of Cytology and Genetics, SB RAS, Lavrentiev av., 10, 630090 Novosibirsk, Russia; pershina@bionet.nsc.ru; 2 Kurchatov Genomics Center, Institute of Cytology and Genetics, SB RAS, Lavrentiev av., 10, 630090 Novosibirsk, Russia; 3 Kurchatov Genomics Center of ARRIAB, All-Russia Research Institute of Agricultural Biotechnology, Timiryazevskaya Street, 42, 127550 Moscow, Russia; divashuk@gmail.com; 4 Moscow Timiryazev Agricultural Academy, Russian State Agrarian University, Timiryazevskaya Street, 49, 127550 Moscow, Russia; irbis-sibri@yandex.ru; 5 Omsk Agricultural Scientific Center, 644012 Omsk, Russia; belan_skg@mail.ru (I.A.B.); rosseeva@mail.ru (L.P.R.)

**Keywords:** alloplasmic lines (*H. vulgare*)-*T. aestivum*, mitochondrial and chloroplast DNA, cytonuclear compatibility, 1BS chromosome arm

## Abstract

The genetic mechanisms of fertility restoration in alloplasmic bread wheat with the barley cytoplasm are poorly explored. The effect of the 1BS chromosome arm on the fertility of bread wheat with the *H. vulgare* cytoplasm was studied depending on the incompleteness/completeness of the cytonuclear compatibility. (i) Three self-fertile (SF) lines and one partially fertile (PF) line with an incomplete cytonuclear compatibility and (ii) four self-fertile (SF) lines with a complete cytonuclear compatibility were studied. For the lines in group (i), the heteroplasmy (simultaneous presence of barley and wheat copies) of the 18S/5S mitochondrial (mt) repeat was revealed as well as the barley-type homoplasmy of chloroplast simple sequence repeats (cpSSRs). In the lines in group (ii), the 18S/5S mt repeat and cpSSRs were found in the wheat-type homoplasmic state. In all of the lines, the 1BS chromosome arm was substituted for the 1RS arm. The F_1_ plants of SF(i)-1BS × 1RS hybrids were fertile. The results of a segregation analysis in the F_2_ plants of SF(i)-1BS × 1RS showed that 1BS carries a single dominant fertility restorer gene (*Rf*) of bread wheat with the *H. vulgare* cytoplasm. All of the F_1_ plants of PF(i)-1BS × 1RS hybrids were sterile. A single dose of this restorer gene is not sufficient to restore fertility in this alloplasmic PF(i) line. All of the F_1_ and F_2_ plants of SF(ii)-1BS × 1RS hybrids were self-fertile.

## 1. Introduction

Alloplasmic lines have been developed as a result of repeated backcrosses of wide hybrids with the pollen parent and by combining the cytoplasm of the maternal parent with the nuclear genome of the paternal parent [1]. As a result of cytoplasm replacement, new nuclear-cytoplasmic interactions arise, which can cause epigenetic modifications of nuclear genes [2]. Depending on the cytoplasm origin and genetic background, alloplasmic lines undergo changes in metabolism [3], sensitivity to stress factors [4,5] and morphological and agronomical traits [2,6,7].

From a practical perspective, alloplasmic lines are produced from different species of cultivated plants and characterized by cytoplasmic male sterility (CMS), which represents one of the three systems used to obtain hybrid seeds [8]. It has been emphasized that the key aspects of the implementation of this CMS-based hybrid production system are the selection of sources of cytoplasms with CMS and *Rf* genes that effectively maintain and restore male fertility.

Cytoplasm replacement is also considered to be an additional source of diversity in cultivated plants [9]. A large number of alloplasmic lines carrying the cytoplasm of wheat relatives have been produced for wheat [1,10]. In fertile alloplasmic lines of bread wheat with the *Aegilops squarrosa* cytoplasm, depending on the genetic backgrounds, early maturation and increased yield were observed, which were explained by nucleus-cytoplasm heterosis [11].

An example of the use of nucleus-cytoplasm heterosis is the development of the bread wheat variety “Xiaoshan 2134” with the *Ae. crassa* cytoplasm, characterized by a high grain quality, high yield and resistance to salinity [12]. The source of the two commercial wheat varieties Roason and Rendezvous was the alloplasmic line VPM1 carrying the *Ae. ventricosa* cytoplasm and genes for resistance to the fungal pathogens *Pch-1* and *Sr38/Lr37/Yr17*, introgressed from *Ae. ventricosa* [13,14]. Fertile lines of bread wheat with *T. timopheevii* and *Secale cereale* cytoplasms, which were characterized by drought tolerance [15] and high-quality gluten, were promising for breeding [16,17]. A few of the recombinant alloplasmic (*H. vulgare*)-*T. aestivum* lines with full fertility restoration and their doubled haploid (DH) lines were used to obtain introgression genotypes [18,19], which were used in breeding [20]. As a result of the selections in different regions, alloplasmic introgression lines L-311(4), L-311(5) and L-311(6) carrying the wheat-rye translocation 1RS.1BL became commercially valuable varieties of spring bread wheat (Uralosibirskaya 2, Sigma and Ishimskaya 11, respectively) [19,20]. In addition, using the L-311(4) line, promising double haploid (DH) lines carrying new complexes of genes for a resistance to fungal pathogens were obtained [21].

According to our data, not only should alloplasmic lines for use in breeding be stable and fertile and have important agronomic traits but, in these alloplasmic lines, the process of cytonuclear compatibility should be complete and disrupted neither during in vitro anther culture nor by the introgression of alien germplasm into the nuclear genome [18,19,21]. Understanding the genetic mechanisms responsible for the restoration of the fertility of alloplasmic genotypes that can be used in breeding is an important and currently unsolved problem. For this reason, the aim of this work was to study the effect of the chromosome arm 1BS on the fertility of alloplasmic recombinant lines (*H. vulgare*)-*T. aestivum*, depending on the degree of completeness of cytonuclear compatibility. The choice of this chromosome was due to the fact that many varieties of bread wheat carry a recombinant chromosome 1RS.1BL consisting of the 1BL arm of wheat and 1RS arm of rye [22] as well as the fact that several *Rf* genes controlling male fertility restoration in wheat with alien cytoplasms are located on the 1B chromosome [23,24] and 1BS arm [25].

## 2. Materials and Methods

### 2.1. Alloplasmic Recombinant Lines (H. vulgare)-T. aestivum

**Figure 1:**
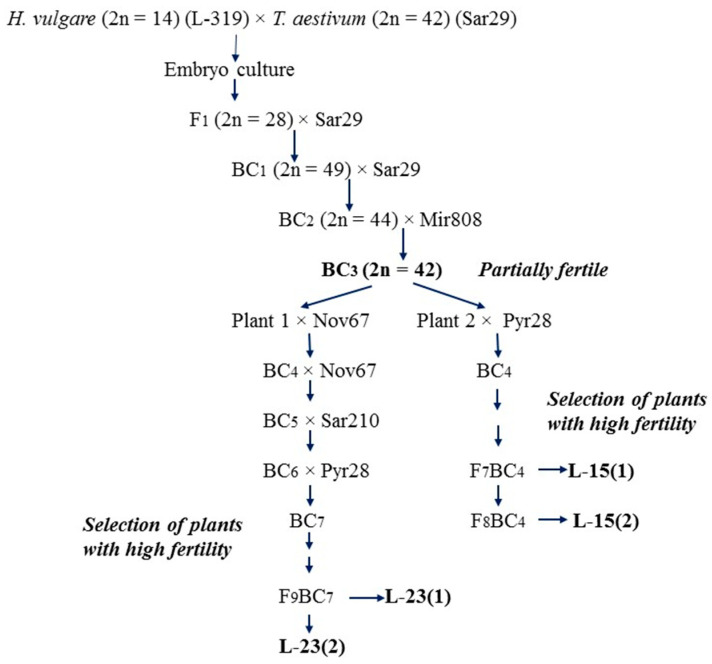
The scheme for the production of alloplasmic recombinant (*H. vulgare*)-*T. aestivum* lines L-15(1), L-15(2), L-23(1), L-23(2). (L-319: barley line; varieties of bread wheat: Sar29: Saratovskaya 29; Mir808: Mironovskaya 808; Pyr28: Pyrotrix 28; Sar210: Saratovskaya 210; Nov67: Novosibirskaya 67).

Eight alloplasmic recombinant lines (*H. vulgare*)-*T. aestivum* were studied and classified into four groups: lines L-15(1), L-15(2) (group 1), lines L-23(1) and L-23(2) (group 2); lines L-55(1) and L-55(2) (group 3), lines L-55(3) and L-55(4) (group 4). Each group included lines of the same origin. Backcrossed progenies of the barley-wheat hybrid *H. vulgare* L. (L-319) × *T. aestivum* L. (var. Saratovskaya 29) were the source of the barley cytoplasm for the alloplasmic lines of groups 1 and 2. As a result of backcrossing with different wheat varieties, partially fertile 42-chromosome plants without barley chromosomes were isolated from BC_3_ plants [26]. Two of these plants, designated as plant 1 and plant 2, were re-pollinated with wheat: plant 1 until BC_7_ generation and plant 2 until BC_4_ generation. The plants of BC_7_ and BC_4_ were self-pollinated and plants with the highest fertility level were selected for the formation of the next self-pollinated generation. The lines L-15(1) and L-15(2) (group 1) were formed from individual fertile plants of the F_7_BC_4_ and F_8_BC_4_ generations, respectively (Figure 1). The lines L-23(1) and L-23(2) (group 2) were formed from individual fertile plants of the F_8_BC_7_ generation (Figure 1).

**Figure 2:**
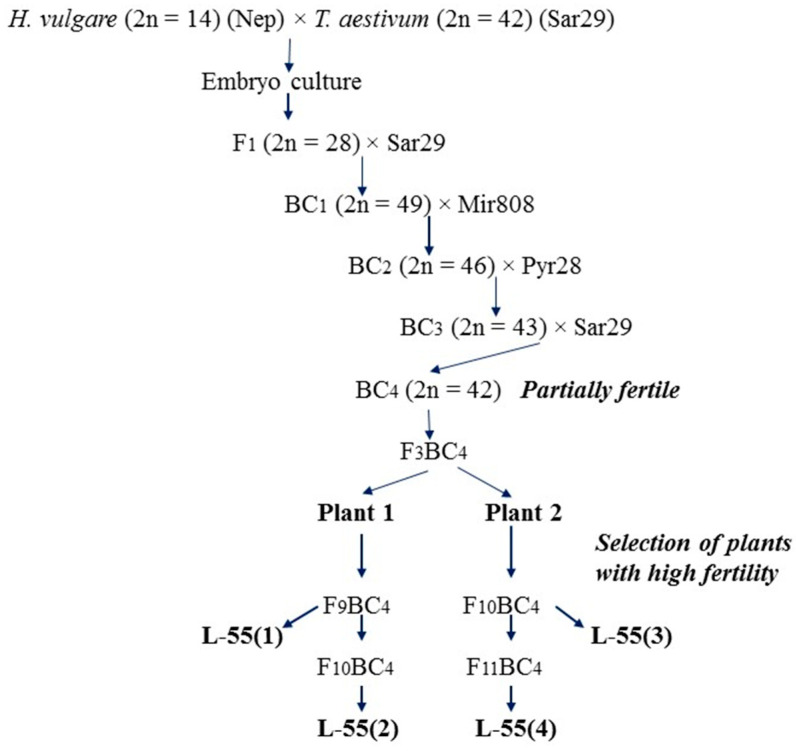
The scheme for the production of alloplasmic recombinant lines (*H. vulgare*)-*T. aestivum* lines L-55(1), L-55(2), L-55(3) and L-55(4). (Nep: barley variety Nepolegaushii; varieties of bread wheat: Sar29: Saratovskaya 29; Mir808: Mironovskaya 808; Pyr28: Pyrotrix 28.

The selection of fertile plants for the production of alloplasmic recombinant lines L-55(1) and L-55(2) (group 3) as well as L-55(3) and L-55(4) (group 4) was carried out starting from the self-pollination of the F_3_BC_4_ generation of the hybrid *H. vulgare* (var. Nepolegaushii) × *T. aestivum* (var. Saratovskaya 29). The plants of F_3_BC_4_ were characterized by a partial fertility and the absence of barley chromosomes [26]. For this work, one of the partially fertile F_3_BC_4_ plants, designated as plant 1, was included for self-pollination when obtaining lines L-55(1) and L-55(2) and plant 2 of the F_3_BC_4_ group was included when obtaining lines L-55(3) and L-55(4). Figure 2 shows the scheme used to obtain the lines of group 3 and group 4. Group 3 consisted of line L-55(1) formed from the fertile plant 1 of F_9_BC_4_ and the line L-55(2) formed from the fertile plant 1 of F_10_BC_4_. Group 4 was represented by line L-55(3) formed from the fertile plant 2 of F_10_BC_4_ and line L-55(3) from the fertile plant 2 of F_11_BC_4_.

In all cases, the source plants for the formation of alloplasmic recombinant lines were selected among self-fertile plants using the 18S/5S mt repeat as a marker for the distinction of alloplasmic lines into lines with cytonuclear incompatibility and compatibility [27,28]. Each group included lines with a close origin but that differed in the incompleteness/completeness of their cytonuclear compatibility (Table 1).

### 2.2. Control Genotypes

As a control, we used the line L-319 of barley *H. vulgare*; the line Om29, isolated from wheat variety Omskaya 29 (Om29), which carries the wheat-rye translocation 1RS.1BL [29]; the line of Saratovskaya 29 (Sar29); the alloplasmic recombinant line L-17(3) and reciprocal F_1_ hybrids obtained by crossing line L-17(3) with the euplasmic (with the wheat cytoplasm) line Om29 (Table 1). The L-17(3) line belongs to the F_14_BC_4_ generation of the barley-wheat hybrid *H. vulgare* L. (var. Nepolegaushii) × *T. aestivum* L. (var. Sar29) [29].

### 2.3. Assessment of the Fertility of Alloplasmic Lines and Hybrids between Alloplasmic Lines and the Line Om29-1RS.1BL

The plants of the alloplasmic recombinant lines and controls were grown in a greenhouse. Spikes from the three main spikes per plant were bagged before flowering and their self-fertility (%) was estimated by dividing the total number of viable seeds in the two lateral florets of each spikelet by the total number of normally developed lateral florets in the spike and multiplying this by 100. The differences between the average values of all studied traits of two alloplasmic lines in each of the four groups were statistically evaluated by a Student’s t-test. Data were analyzed using Statistica v.7.0.61.0. In addition, the frequency (%) of fertile plants was determined for each line. At least 20 plants of each line were used in each treatment.

To study the effect of the chromosome 1BS on the fertility of the alloplasmic lines (*H. vulgare*)-*T. aestivum* depending on the degree of cytonuclear compatibility, lines L-15(1), L-15(2), L-23(1), L-23(2), L-55(1), L-55(2), L-55(3) and L-55(4) were crossed as maternal genotypes with the line Om29-1RS.1BL. The fertility was studied in F_1_ and F_2_ plants grown in a greenhouse. A plant was considered male-sterile if it contained no seeds whereas it was considered male fertile if it contained at least one seed. A chi-squared test (α = 0.05) for the goodness of fit was used for the deviation of the observed data from the theoretically expected segregation in F_2_.

### 2.4. Studying of mt and cpDNA Regions

Total DNA from two one-week old seedlings (at least five from each line) was isolated according to the previously published protocols [30]. The mitochondrial 5′ upstream region of the 18S/5S repeat was amplified by PCR using specific primers (F 5′-TTCTCGCGTTCCCTTAATTC-3′; R 5′-CGTTCGCCACTTTGTTCTCA-3′) using the following program: an initial denaturation step at 94 °C for 4 min, 35 cycles of denaturation at 92 °C for 20 s, annealing at 45 °C for 30 s and extension at 72 °C for 2 min followed by a final extension at 72 °C for 10 min [27]. Specific primers for the 18S/5S repeat were designed based on the mitochondrial genome sequences published in Coulthart et al. [31]. The PCR products were electrophoresed in a 1.5% agarose gel with 1 × TAE buffer.

To identify whether the chloroplast DNA in the studied lines belonged to wheat or barley, markers for the microsatellite regions of chloroplast DNA TaCM4 and TaCM9, developed in Tomar et al. [32], were used. A few modifications were made in the primers for the complete amplification from barley cpDNA (Table 2).

A PCR was performed in a 25 μL reaction volume containing 70 mM Tris-HCl buffer (pH 8.6), 16.6 mM (NH_4_)_2_SO_4_, 2.5 mM MgCl_2_, 0.2 mM of each dNTP, 10% *v/v* dimethyl sulfoxide, 0.3 μM forward and reverse primers (Sintol Ltd., Moscow, Russia), 1.25 U of colored Taq-polymerase (Sileks Ltd., Moscow, Russia) and 100 ng of template DNA. The cycling conditions involved an initial denaturation at 94 °C for 3 min followed by 35 cycles of denaturation at 94 °C, primer annealing at 60 °C and a primer extension at 72 °C, each for 30 s. A final extension at 72 °C for 5 min was carried out and the PCR products were stored at 4 °C until electrophoresis. The PCR was performed in a GeneAmp PCR System 9700 (Applied Biosystems, Foster City, CA, USA). The PCR products were separated in a 2% agarose gel in a TBE buffer using a GeneRuler 100 bp DNA Ladder (Thermo Fisher Scientific, Waltham, MA, USA) as a molecular weight marker and stained with ethidium bromide for a subsequent visualization in Gel Doc XR+ (Bio-Rad Laboratories, Inc., Hercules, CA, USA).

The control in the study of alloplasmic recombinant lines consisted of line L-319 of cultivated barley, one of the sources of the cytoplasm of alloplasmic lines the bread wheat varieties Saratovskaya 29 (one of paternal genotypes) and Omskaya 29; alloplasmic recombinant line L-17(3) and reciprocal hybrids L-17(3)/Omskaya 29 and Omskaya 29/L-17(3).

### 2.5. Identification of 1RS Chromosome Arm

To identify the presence of the 1RS chromosome arm in the hybrids and to differentiate plants that were homozygous for the 1BL.1RS from the heterozygous plants, we used a codominant PCR marker including two pairs of primers: one from the wheat Glu-B3 gene and the other from the rye ω-secalin gene [33]. The primer sequences were as follows: ω-sec-P1 (ACCTTCCTCATCTTTGTCCT) and ω-sec-P2 (CCGATGCCTATACCACTACT); O11B3 (GTTGCTGCTGAGGTTGGTTC) and O11B5 (GGTACCAACAACAACAACCC) (locus: Glu-B3).

## 3. Results

### 3.1. Study of the Level of Fertility of Alloplasmic Lines

In all lines except for line L-55(1), all of the plants were fertile. In line L-55(1), only 85% of the plants were fertile. This line was classified as partially fertile. As for self-fertility (%), lines L-15(2), L-23(2) and L-55(2) exceeded the lines from their groups, L-15(1), L-23(1) and L-55(1) in this indicator. Lines L-55(3) and L-55(4) (group 4) did not differ in this indicator (Table 3). The value of self-fertility (%) in the alloplasmic recombinant line L-17(3) did not differ from that in reciprocal hybrids L-17(3)/Om29 and Om29/L-17(3).

### 3.2. PCR Analysis of 18S/5S mtDNA Repeat in Alloplasmic Recombinant and Introgression Lines (H. vulgare)-T. aestivum

**Figure 3:**
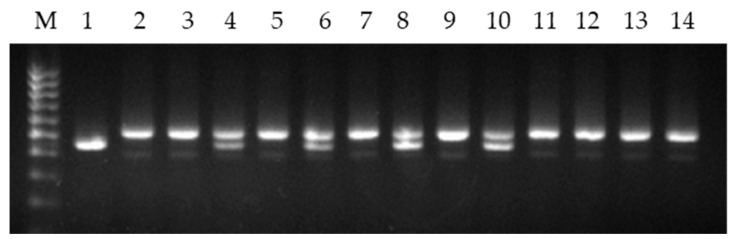
Agarose gel electrophoresis of PCR products from alloplasmic lines using the 18S/5S mt repeat. M: 100 bp ladder marker; 1: L-319; 2: Om29; 3: Sar29; 4: L-15(1); 5: L-15(2); 6: L-23(1); 7: L-23(2); 8: L-55(1); 9: L-55(2); 10: L-55(3); 11: L-55(4); 12: L-17(3); 13: L-17(3)/Om29; 14: Om29/L-17(3).

The primer combination for the 5′ upstream region of 18S/5S has previously been shown to amplify different-sized mtDNA fragments between wheat and barley [27,28]. The study of the 18S/5S repeat in two pairs of each group of alloplasmic lines showed that they differed by parental types of mtDNA. In lines L-15(1), L-23(1), L-55(1) and L-55(3), a heteroplasmy was revealed (the simultaneous presence of barley and wheat mtDNA copies) (Figure 3). In lines L-15(2), L-23(2), L-55(2) and L-55(4), only wheat mtDNA copies were found.

The control group, when studying the lines, included the line L-319 of barley *H. vulgare*, one of the sources of the cytoplasm of alloplasmic lines, and the bread wheat (*T. aestivum*) varieties Saratovskaya 29 (one of paternal genotypes) and Omskaya 29. In addition, the alloplasmic recombinant line L-17(3) and reciprocal hybrids L-17(3)/Om29 and Om29/L-17(3) were studied as controls. In these control variants, as well as in wheat varieties, only wheat mtDNA copies were detected. The data of this part of the work are given in Table 3.

The PCR analysis of the 18S/5S mt repeat was used to select the source plants of fertile alloplasmic lines with an incomplete and a complete cytonuclear compatibility. The lines with an incomplete cytonuclear incompatibility included plants in which both barley and wheat copies of the 18S/5S mt repeat were revealed. Plants with only wheat-type 18S/5S repeat copies were classified as lines with a complete cytonuclear compatibility.

### 3.3. Analysis of cpDNA Microsatellite Loci in Alloplasmic Recombinant and Introgression Lines (H. vulgare)-T. aestivum

**Figure 4:**
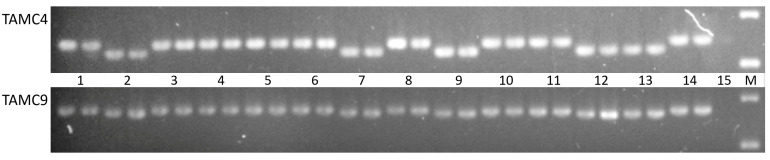
Amplified SSR markers TaCM4 and TaCM9 separated in 2% agarose gel electrophoresis. 1: L-15(2); 2: L-15(1); 3: L-17; 4: L-17(3)/Om29; 5: Om29/L-17(3); 6: L-55(2); 7: L-55(1); 8: L-55(4); 9: L-55(3); 10: wheat variety Omskaya 29; 11: L-23(2); 12: L-23(1); 13: barley L-319; 14: wheat variety Saratovskaya 29; 15: negative control (distilled water); M: GeneRuler 100 bp DNA Ladder.

The comparative analysis of chloroplast DNA microsatellite loci in parental accessions of wheat (Saratovskaya 29) and barley (L-319) revealed differences only in two molecular markers: TaCM4 and TaCM9. The size of the obtained products using these markers was consistent with those expected based on the nucleotide sequences of wheat and barley in the NCBI. The analysis of the alloplasmic lines using TaCM4 and TaCM9 demonstrated completely identical results for both markers. Wheat-type amplification for both markers was observed in lines L-15(2), L-55(2), L-55(4), L-23(2) and L-17(3) and hybrids L-17(3)/Om29 and Om29/L-17(3). The amplicon size in lines L-15(1), L-55(1), L-55(3) and L-23(1) corresponded to products obtained in barley cpDNA (Table 3, Figure 4).

### 3.4. Analysis of Hybrids Derived from Crosses between Alloplasmic Lines and the Line Om29-1RS.1BL

Alloplasmic lines L-15(1), L-15(2), L-23(1), L-23(2), L-55(1), L-55(2), L-55(3) and L-55(4) were used as female parents and crossed with the line Om29-1RS.1BL as a male parent to generate F_1_ seeds. Furthermore, at least 10 F_1_ plants were grown in a greenhouse, the spikes of which were bagged before flowering. The number of F_1_ plants that set seeds from self-pollination was determined. It was found that all F_1_ plants of hybrid combinations, except for L-55(1) × Om29-1RS.1BL, were fertile. All F_1_ plants of the hybrid combination L-55(1) × Om29-1RS.1BL were sterile (Table 4). This indicated that the fertility of the alloplasmic recombinant line L-55(1) depended on the chromosome 1BS and one dose of the gene localized on the 1BS arm was not sufficient to restore the fertility of this line. The study of seed setting in 108 F_2_ plants of the hybrid combination L-15(1) × Om 29-1RS.1BL showed that the total number of fertile plants was 74 and the number of sterile plants was 34. The observed frequency of plants fitted well with the expected segregation ratio of 3 (fertile):1 (sterile) with an χ^2^ value of 2.42 (*p* value = 0.120) at a 5% level of significance (Table 4). Similar results were obtained for two other hybrid combinations L-23(1) × Om29-1RS.1BL and L-55(3) × Om29-1RS.1BL. Thus, the observed frequency of 76 fertile and 21 sterile plants in the F_2_ population showed a good fit with the Mendelian segregation ratio of 3 (fertile):1 (sterile) with an χ^2^ value of 0.58 (*p* value = 0.446) at a 5% level of significance (Table 4). In the hybrid combination L-55(3) × Om29-1RS.1BL, the total number of fertile F_2_ plants was 58 and the number of sterile plants was 17. Thus, segregation in the F_2_ of hybrid L-55(3) × Om29-1RS.1BL fitted a 3 (fertile):1 (sterile) ratio with an χ^2^ value of 0.22 (*p* value = 0.640) at a 5% level of significance (Table 4). These results showed the dependence of fertility in alloplasmic recombinant lines L-15(1), L-23(1) and L-55(3) on the 1BS chromosome arm. The segregation ratios in the F_2_ population of these lines using data on seed setting indicated that the fertility restoration was controlled by a single dominant restorer gene (*Rf*) located on this 1BS chromosome arm.

Other results were obtained for the hybrid combinations L-15(2) × Om29-1RS.1BL; L-23(2) × Om29-1RS.1BL; L-55(2) × Om29-1RS.1BL and L-55(4) × Om29-1RS.1BL. All 86 F_2_ plants of L-15(2) × Om29-1RS.1BL, 84 F_2_ plants of L-23(2) × Om29-1RS.1BL, 66 F_2_ plants of L-55(2) × Om29- 1RS.1BL and 96 F_2_ plants of L-55(4) × Om29-1RS.1BL were fertile.

### 3.5. Identification of the 1RS Chromosome Arm in F_1_ and F_2_ Hybrids Derived from Alloplasmic Lines and the Om29-1RS.1BL Line Crosses

**Figure 5:**
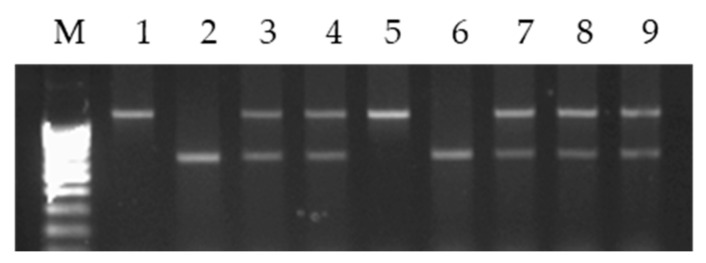
Multiplex PCR detection of 1BL.1RS translocation with a codominant marker. M: 100 bp ladder marker. 1: Om29-1RS.1BL; 2: Sar29; 3: F_1_ of L-15(1) × Om29-1RS.1BL; 4: F_2_ of L-15(1) × Om29-1RS.1BL; 5: F_2_ of L-15 (1) × Om 29-1RS.1BL (homozygous for the 1RS.1BL); 6: F_2_ of L-15 (1) × Om 29-1RS.1BL; 7: F_2_ of L-23(1) × Om 29-1RS.1BL; 8: F_2_ of L-55(1) × Om 29-1RS.1BL; 9: F_2_ of L-55(3) × Om 29-1RS.1BL (heterozygotes for 1RS.1BL).

The selective analysis of individual F_1_ samples of all hybrid combinations showed the presence of the rye ω-secalin gene, which confirmed the presence of the 1RS chromosome arm in the F_1_ hybrids. By combining the PCR assay resulting in the 1.1-kb fragment from the 1RS arm and a PCR assay resulting in a 0.6 kb fragment from the *Glu-B3* gene on the 1BS arm, plants homozygous for the 1RS.1BL line were distinguished from the heterozygous plants by segregating the F_2_ population. A selective analysis using this codominant marker showed that sterile plants were homozygous for 1RS and, among fertile plants, there were heterozygotes plants for 1RS and homozygotes plants for 1BS. The results of the analysis of a few samples in F_1_ and F_2_ are shown in Figure 5.

## 4. Discussion

Earlier in our work, we developed and studied alloplasmic recombinant lines (*H. vulgare*)-*T. aestivum* with different origins of three main types: (1) sterile, (2) partially fertile (segregated into partially fertile and sterile plants) and (3) stably self-fertile lines [18,27,28]. Based on the PCR analysis of the 18S/5S mt repeat, it was possible to classify them into two types depending on the incompleteness of the cytonuclear compatibility (sterile and partially fertile) and the completeness of the cytonuclear compatibility (stably self-fertile). In plants of sterile and partially fertile lines, barley and wheat copies of the 18S/5S mt repeat were simultaneously detected (heteroplasmy) and in plants of stably self-fertile lines, only wheat-type copies (homoplasmy) were detected [27,28].

Theoretically, when alloplasmic lines are obtained by backcrossing hybrids with the paternal genotype, a strictly maternal inheritance of organelle genomes occurs. However, in wide crosses, the mechanism of organellar DNA transmission can be disrupted through a replacement with a paternal [34] or biparental inheritance [35,36,37]. A biparental inheritance leads to heteroplasmy (the presence in plant cells of more than one organellar DNA variant), which has been revealed in different hybrid combinations of wheat in F1 and their backcross progenies [27,28,36,37]. In alloplasmic lines (*H. vulgare*)-*T. aestivum* barley chromosomes were undetected and apparently replaced by wheat chromosomes; a level of variability of mitochondrial and chloroplast DNA was observed, associated with either plant sterility or fertility restoration [18,27,28]. Thus, in partially fertile alloplasmic lines, or when their sterility was fixed due to cytonuclear incompatibility, the copies of the barley organellar DNA prevailed. The restoration of fertility during repeated backcrosses with wheat was accompanied by the selective amplification of wheat organellar DNA copies [27]. The same patterns of variability of the mitochondrial regions were observed during the development of alloplasmic wheat lines carrying the cytoplasm of the *Aegilops* species [35]. In this work, we studied four groups of fertile alloplasmic recombinant lines (*H. vulgare*)-*T. aestivum*, which were classified using 18S/5S mt repeat analysis data on lines with an incomplete and a complete cytonuclear compatibility. Each group included two lines of the same origin but they differed in the degree of completeness of cytonuclear compatibility. The lines were characterized by fertility, the results of the cpDNA analysis and the effect of the 1BS chromosome arm on the fertility depending on the completeness of cytonuclear compatibility. All of the lines except for L-55(1) from group 3 showed a stable self-fertility. The L-55(1) line showed a segregation into fertile and sterile plants so it was classified as partially fertile. The data obtained during the comparative analysis of the morphological characteristics of the lines within the groups showed that these characteristics were not in all cases a criterion for distinguishing lines with an incomplete and a complete cytonuclear compatibility.

The results of the analysis of the cpDNA regions showed differences between the lines with an incomplete and a complete cytonuclear compatibility. Thus, only barley-type copies of cpDNA were found in self-fertile plants of lines with an incomplete cytonuclear compatibility. Only wheat-type cpDNA copies were detected in the self-fertile plants of lines with a complete cytonuclear compatibility. These results were consistent with the data of our previous works on the study of other regions of cpDNA in alloplasmic lines (*H. vulgare*)-*T. aestivum* of a different origin [18,27,28]. Thus, the results of the cpDNA analysis confirmed the classification of the studied alloplasmic recombinant lines to a certain type of cytonuclear compatibility.

Apparently, in the stable self-fertile lines, a newly formed nuclear recombinant genome, from which barley chromosomes had been eliminated, affected the change in the initial heteroplasmic state of the organelle genomes due to the biparental inheritance in hybrids towards wheat-type homoplasmy as a more suitable condition for restoring cytonuclear compatibility.

This variant of events was defined as the loss of the initial heteroplasmic state [27]. In partially fertile lines, this process had not yet been completed and therefore we observed mtDNA heteroplasmy and maternal-type cpDNA homoplasmy, which might have been a consequence of transient cpDNA heteroplasmy in previous generations of hybrids. This assumption was consistent with the data on changes in the quantitative ratio of heteroplasmic variants, which can occur rather quickly and lead to the predominance of a certain mitotype in subsequent generations [38].

Clear differences in the effect of the 1BS chromosome arm on fertility were found in alloplasmic recombinant lines with a different cytoplasmic background. In lines with an incomplete cytonuclear compatibility, the 1BS arm affected plant fertility while in lines with a complete cytonuclear compatibility, it did not.

Based on the results of the fertility segregation in F_2_ hybrids obtained from the crossing of three alloplasmic recombinant lines with the line Om29-1RS.1BL, it could be concluded that the 1BS arm carried a dominant monogenic restorer-of-fertility *(Rf)* gene that was responsible for the fertility restoration of bread wheat with the barley cytoplasm in the studied lines with an incomplete cytonuclear compatibility. However, a single dose of this *Rf* gene was not sufficient to restore the fertility of the alloplasmic partially fertile L-55(1) line. This conclusion was based on the fact that F_1_ hybrids L-55(1) × Om29-1RS.1BL, heterozygous for the 1BS arm, were completely sterile. The disruption of the nuclear-mitochondrial interactions in plants by an alloplasmic condition usually leads to cytoplasmic male sterility (CMS) [39]. CMS is associated with mutations in the mitochondrial genes that negatively affect the target nuclear genes responsible for the development of flower organs and pollen, causing sterility [40]. Male fertility can be restored by the expression of nuclear *Rf* genes by a strong reduction in the production of mitochondrial CMS-inducing proteins [41]. The most well-studied systems of the restoration of male fertility of wheat with an alien cytoplasm are the alloplasmic wheat lines carrying the *T. timopheevii* cytoplasm. To date, nine *Rf* genes (*Rf1*–*Rf9*) have been reported to restore fertility against the *T. timopheevii* cytoplasm and have been located on different wheat chromosomes [24]. *Rf3* is one of the major genes and was localized on the chromosome 1B [23,24]. Another *Rf* gene, called *Rf ^multi^*, was also located on the chromosome 1BS and found to be effective in the cytoplasms of *Aegilops kotschyi*, *Ae. mutica* and *Ae. uniaristata* [25]. Here, we reported the identification of the *restorer-of-fertility* (*Rf*) gene on the 1BS chromosome arm, which controls the fertility restoration of bread wheat with the *H. vulgare* cytoplasm.

The alloplasmic recombinant lines (*H. vulgare*)-*T. aestivum* with an incomplete nuclear-cytoplasmic compatibility studied in our work were considered as models for the further localization of other restorer genes (*Rf*) in bread wheat with the *H. vulgare* cytoplasm.

Wheat varieties Saratovskaya 29, Mironovskaya 808 and Pyrotrix 28 were used for the formation of the recombinant nuclear genome of all alloplasmic lines studied in this work. In addition, when obtaining lines L-23(1) and L-23(2), the varieties Novosibirskaya 67 (twice) and Saratovskaya 210 were included in the backcrosses (Table 1). The variety Saratovskaya 29 as a pollen parent crosses better with barley than other wheat genotypes and when using an embryo rescue of hybrid combinations of barley and Saratovskaya 29, viable barley-wheat hybrids developed, which, although they were male-sterile, exhibited female fertility [42]. This made it possible to include barley-wheat hybrids in backcrosses with bread wheat in order to restore fertility in the backcross progenies.

However, the variety Saratovskaya 29 was found to be a fixer for the sterility of bread wheat with the cytoplasm of *H. vulgare*: as a result of backcrosses of barley-wheat hybrids with this variety, a complete sterility was established by BC_5_–BC_8_ generations [42]. The introduction of the varieties Mironovskaya 808 [19] and Pyrotrix 28 [42] into backcrossing led to the fertility restoration of plants of the backcross generations BC_3_–BC_4_, which made it possible to obtain alloplasmic lines.

Using an SSR analysis, it was shown that the differences in the level of fertility between the alloplasmic recombinant lines were determined by the different amounts of chromatin of the varieties Saratovskaya 29 and Pyrotrix 28. The full fertility restoration in alloplasmic recombinant lines was accompanied by the formation of a nuclear genome in which the chromatin of Pyrotrix 28 prevailed [42]. It was concluded that the variety Pyrotrix 28 was a carrier of genes that determined the restoration of bread wheat fertility with the *H. vulgare* cytoplasm. Apparently, the different chromatin ratio of wheat varieties in recombinant nuclear genomes determined the different level of fertility in the partially fertile line L-55(1) and related fertile lines L-55(2), L-55(3) and L-55(4). It could be assumed that the variety Novosibirskaya 67 also had a positive effect on the fertility of the alloplasmic lines (*H. vulgare*)-*T. aestivum* or at least it did not have a negative effect as this variety was twice used as a recurrent parent when creating the fertile alloplasmic recombinant line L-23(2).

Earlier, on the basis of alloplasmic recombinant lines that were sister lines of L-17(3) studied in this work as a control, DH lines were obtained, which were used as maternal lines to obtain introgression genotypes for breeding [19]. Using one of these DH lines, three commercial varieties of spring bread wheat were developed that carry the wheat-rye translocation 1RS.1BL with the gene complex *Lr26/Sr31/Yr9/Pm8* controlling the resistance to fungal pathogens [19,20] as well as promising breeding lines combining 1RS.1BL translocation with genes for a resistance to fungal pathogens from different sources [20,21]. The data obtained in this work confirmed that the substitution of the 1BS chromosome arm for the 1RS arm in the genome of alloplasmic recombinant lines (*H. vulgare*)-*T. aestivum* with a complete nuclear-cytoplasmic compatibility did not lead to male sterility.

## 5. Conclusions

In this study, the differences between fertile alloplasmic recombinant lines (*H. vulgare*)-*T. aestivum* with an incomplete and a complete cytonuclear compatibility were studied. Pairs of fertile lines of the same origin but that differed from each other in the degree of completeness of cytonuclear compatibility were isolated on the basis of 18S/5S mt repeat analysis data among self-pollinated progenies of partially fertile plants of backcross generations obtained as a result of backcrosses of barley-wheat hybrids with different varieties of bread wheat. Clear differences between the alloplasmic lines of these two groups were found in the parental types of the 18S/5S mt repeat, cpDNA regions and influence of the 1BS chromosome arm. The heteroplasmy (simultaneous presence of barley and wheat copies) of the 18S/5S mitochondrial repeat was revealed as well as the barley-type homoplasmy of chloroplast SSRs in lines with an incomplete cytonuclear compatibility. Lines with an incomplete cytonuclear compatibility turned out to be convenient experimental models for identifying *restorer-of-fertility* (*Rf*) genes, which are responsible for the fertility restoration of bread wheat carrying the *H. vulgare* cytoplasm.

## Figures and Tables

**Table 1:** Origin of alloplasmic recombinant (*H. vulgare*)-*T. aestivum* lines and control genotypes. (*H.v*.: *H. vulgare*; *T.a.*: *T. aestivum*; L-319: line of barley; Nep: barley variety Nepolegaushii; wheat varieties: Sar29: Saratovskaya 29; Sar 210: Saratovskaya 210; Mir808: Mironovskaya 808; Pyr28: Pyrotrix 28; Nov67: Novosibirskaya 67.

Groups	Lines	Origin of Lines	Generation
1	L-15(1)	[*H.v.* (L-319) × *T.a.* (Sar29)]/Sar29/Mir808/Pyr28	F_9_BC_4_
	L-15(2)		
2	L-23(1)	[*H.v.* (L-319) × *T.a.* (Sar29)]/Sar29/Sar29/Mir808/Nov67/Nov67/Sar210/Pyr28	F_7_BC_7_
	L-23(2)		F_8_BC_7_
3	L-55(1)	[*H.v.* (Nep) × *T.a.* (Sar29)]/Sar29/Mir808/Pyr28/Sar29	F_9_BC_4_
	L-55(2)		F_10_BC_4_
4	L-55(3)		F_10_BC_4_
	L-55(4)		F_11_BC_4_
Controls	L-17(3)	[*H.v.* (Nep) × *T.a.* (Sar29)]/Mir808/Mir808/Sar29	F_14_BC_3_
	L-17(3)/Om29 Om29/L-17(3)	L-17(3)/Omskaya 29 Omskaya 29/L-17(3)	F_1_
	L-319 Om29 Sar29	*H. vulgare*, line L-319 *T. aestivum,* var. Omskaya 29 *T. aestivum,* var. Saratovskaya 29	

**Table 2:** Details of the simple sequence repeat markers for the wheat and barley chloroplast genome (TA: *T. aestivum*; HV: *H. vulgare*).

Marker ID	Primer Sequence	Expected Size of the Product, bp
TaCM4	AATCCTTGGGGTTCCAGAAT GCCACTTKGATTTCCCATTA	TA: 242 HV: 224
TaCM9	TCCAGCCAACGATGACACTA CCAAGAAAGCACATCAGATCA	TA: 269 HV: 261

**Table 3:** Results of the study of fertility and mt and cpDNA regions in alloplasmic recombinant lines (*H. vulgare*)-*T. aestivum*. (B: barley; W: wheat; the difference compared with the other line of its group is significantly higher when * *p* < 0.05; *** *p* < 0.001, Student’s *t*-test).

Groups	Lines	Self-Fertility (%)	18S/5S mtDNA Region	SSR cpDNA Loci	
				TaCM4	TaCM9
1	L-15(1) L-15(2)	76, 38 92, 11 ***	B + W W	B W	B W
2	L-23(1) L-23(2)	88, 25 94, 72 *	B + W W	B W	B W
3	L-55(1) L-55(2)	67, 05 87, 80 *	B + W W	B W	B W
4	L-55(3) L-55(4)	90, 83 89, 81	B + W W	B W	B W
Controls	L-17(3) 17(3)/Om29 Om29/17(3)	92, 90 92, 04 89, 41	W W W	W W W	W W W
	L-319 Om29 Sar29	100 98, 11 100	B W W	B W W	B W W

**Table 4:** Segregation for seed setting in F_2_ hybrids derived from the cross of alloplasmic recombinant lines (*H. vulgare*)-*T. aestivum* with Omskaya 29 carrying the wheat-rye translocation 1RS.1BL.

Hybrid Combination	Gene-ration	Total Number of Plants	Seed Set in F_1_ and F_2_ Plants		Expected Segregation Ratio in F_2_	χ^2^	*p* Value
			No. of Fertile Plants	No. of Sterile Plants			
L-15(1) × Om29-1RS.1BL	F_1_	12	12	-	-	-	-
L-15(1) × Om29-1RS.1BL	F_2_	108	74	34	3:1	2.42	0.12
L-15(2) × Om29-1RS.1BL	F_1_	16	16	0	-	-	-
L-15(2) × Om29-1RS.1BL	F_2_	86	86	-	-	-	-
L-23(1) × Om29-1RS.1BL	F_1_	10	10	0	-	-	-
L-23(1) × Om29-1RS.1BL	F_2_	97	76	21	3:1	0.58	0.446
L-23(2) × Om29-1RS.1BL	F_1_	10	10	0	-	-	-
L-23(2) × Om29-1RS.1BL	F_2_	84	84	0	-	-	-
L-55(1) × Om29-1RS.1BL	F_1_	60	0	60	-	-	-
L-55(2) × Om29-1RS.1BL	F_1_	20	20	0	-	-	-
L-55(2) × Om29-1RS.1BL	F_2_	66	66	0	-	-	-
L-55(3) × Om29-1RS.1BL	F_1_	38	38	0	-	-	-
L-55(3) × Om29-1RS.1BL	F_2_	75	58	17	3:1	0.22	0.64
L-55(4) × Om29-1RS.1BL	F_1_	15	15	0	-	-	-
L-55(4) × Om29-1RS.1BL	F_2_	96	96	-	-	-	-
